# Effects of disease-modifying drugs on serum neurofilament light chain, chitinase-3-like-1 protein levels, and selected plasmacytoid dendritic cell biomarkers in relapsing-remitting multiple sclerosis

**DOI:** 10.4102/ajlm.v15i1.2901

**Published:** 2026-01-21

**Authors:** Dalia T. Kamal, Sohair K. Sayed, Tarek A. Rageh, Basant Rashad, Eman R. Badawy

**Affiliations:** 1 Department of Clinical Pathology, Faculty of Medicine, Assiut University, Assiut, Egypt; 2 Department of Neuropsychiatry, Faculty of Medicine, Assiut University, Assiut, Egypt

**Keywords:** multiple sclerosis, disease-modifying drugs, neurofilament light chain, chitinase 3-like 1, plasmacytoid dendritic cells, CD303, CD274, CCR7

## Abstract

**Background:**

Multiple sclerosis (MS) is a neurodegenerative central nervous system disorder causing axonal damage and disability. Relapses develop over hours or days and then subside over weeks. Disease-modifying drugs (DMDs) influence disease activity. Interferon beta-1A (IFN-b-1A) is a widely used first-line treatment for relapsing remitting MS (RRMS) that reduces central nervous system inflammation. Fingolimod affects lymphocyte trafficking. B-cell therapy (rituximab) depletes circulating CD20+ B cells in cerebrospinal fluid, but their specific effects in RRMS remain limited.

**Objective:**

The aim of the present study was to evaluate the effect of DMDs such as IFN-b-1A, fingolimod and rituximab on neurofilament light chain (NFL) and chitinase 3-like 1 (CHI3L1) serum levels, and some biomarkers of plasmacytoid dendritic cells (pDCs) in RRMS patients.

**Methods:**

Thirty healthy controls and 44 RRMS patients actively receiving their DMDs, were recruited into this study. Patients were divided into three groups according to DMDs type: Group 1 (*n* = 17) received IFN-b-1A, Group 2 (*n* = 20) received fingolimod, and Group 3 (*n* = 7) received rituximab. Patients of all ages and both sexes were included.

**Results:**

Serum NFL (84.1% sensitivity and 60.0% specificity) and CHI3L1 (90.9% sensitivity and 73.0% specificity) levels were higher in patients than in controls (*p* ≤ 0.001), with higher levels of NFL in the B-cell therapy group compared with IFN-b-1A (*p* ≤ 0.001) and fingolimod (*p* = 0.005), and higher levels of CHI3L1 in the B-cell group compared to IFN-b-1A (*p* = 0.001) and fingolimod (*p* = 0.015). Plasmacytoid dendritic cells showed no difference in tolerogenic and migratory function between the DMDs groups.

**Conclusion:**

Disease-modifying drug type (IFN-b-1A, fingolimod, and B-cell therapy) impacts NFL and CHI3L1 serum levels as drug response biomarkers and relates to clinical data of MS patients, but has no diverse impact on the migratory and tolerogenic function of pDCs.

**What this study adds:**

The serum NFL and CHI3L1 need to be validated as drug response biomarkers in RRMS patients, evaluating the DMDs’ effect on immunocellular level by studying migratory and tolerogenic functions of pDCs.

## Introduction

Multiple sclerosis (MS) is a degenerative illness of the central nervous system (CNS) that results in demyelination and axonal injury resulting from the migration of peripherally activated immune cells into the CNS.^1^ Both T- and B-cell reactivity to shared infectious and self-antigens (molecular mimicry) and nonspecific activation during infections (bystander activation) are key mechanisms driving MS-related immune activation, blood–brain barrier migration, CNS infiltration, and tissue damage.^2^ Clinically isolated syndrome often indicates MS, usually with optic neuritis, brainstem, or spinal cord syndromes. Primary progressive MS, in 5% to 15% of cases, presents as gradual spastic paraparesis with single-system impairment.^3^ In early relapsing remitting MS (RRMS), relapse recovery often appears complete, despite underlying damage. As reserve declines, recovery lessens, leading to disability, and within 10 to 15 years, many progress to secondary progressive MS.^4^

The Middle East North Africa region falls in the low-to-moderate MS prevalence zone, but there is a trend toward increased MS prevalence over the last few decades, with a rate in the range of 30/100 000 – 84/100 000.^5^ According to the Middle East and North Africa Committee for Treatment and Research in Multiple Sclerosis (Middle East and North Africa Committee for Treatment and Research in Multiple Sclerosis) registry, MS onset was relapsing in 96% and progressive in 3.2% of cases.^6^ The exact MS prevalence in Egypt is unknown because of a lack of national surveys, but a retrospective meta-analysis across 10 referral centres estimated it at 1.41% (14.1 per 1000 neurological cases).^7^

In RRMS, symptoms develop gradually over days and last at least 24 h without fever or infection. A common first sign is unilateral optic neuritis with vision loss, eye pain, and altered colour vision. Vision loss stabilises within two weeks, but recovery may take longer and may be incomplete. Partial spinal cord myelitis causes gradual limb sensory and motor symptoms, with upper motor neuron signs such as spasticity, pyramidal weakness, hyperreflexia, and extensor plantar responses.^8^

Diagnosis of MS is clinical, based on history and examination, supported by investigations. The McDonald Criteria guide diagnosis requires dissemination in time and dissemination in space. Oligoclonal bands in cerebrospinal fluid (CSF) can indicate dissemination in time, supporting RRMS diagnosis in clinically isolated syndrome patients with magnetic resonance imaging (MRI) evidence of dissemination in space.^9^

The initiation of high-efficacy disease-modifying drugs (DMDs) may diminish disease activity, but there are concerns about their varying effects; therefore, fluid biomarkers that can forecast early disease activity following drug initiation may be beneficial.^10^

Loss of axons in MS impacts all types of fibres, resulting in as much as a 60% reduction in the spinal cord. This loss contributes to disability, cognitive deterioration, fatigue, shrinkage of the brain and cervical region, and inadequate response to treatment. Neurofilament light chain (NFL), which is released following axonal damage, can be found in CSF and blood, acting as a marker for disease progression and treatment effectiveness.^11^

Chitinase 3-like 1 (CHI3L1) drives CNS inflammation by activating microglia, macrophages, and astrocytes, promoting cytokine release, blood–brain barrier disruption, and demyelination. It also induces neuronal apoptosis and inhibits neurite growth. As a key mediator of neuroinflammation and neurodegeneration, CHI3L1 is a promising therapeutic target in MS.^12^

The initiation, direction, and inhibition of CNS damage in MS are all roles of dendritic cells (DCs) that control T-cells, provoke an immune response, and ensure that the CNS immune system remains under surveillance. Classical DCs contribute to disease progression, but plasmacytoid dendritic cells (pDCs), which synthesise interferons, play a role in T-regulatory cell maturation and the resolution of illness.^13^ Plasmacytoid DCs represent a small subset of cells characterised by the expression of CD123, CD11c–, HLA-DR+, and express the blood DC antigen BDCA-2 (CD303) and BDCA-4 (CD304) antigens.^14^ Dendritic cells upregulate the expression of CCR7 and CXCR4, which enables them to migrate to lymph nodes, where they can present antigens to naïve T-cells, driving their polarisation toward pro-inflammatory T-cells.^15^ Dendritic cells with a stable, semi-mature phenotype and tolerogenic attributes are designated as tolerogenic DCs. Many tolerogenic DCs express programmed death-ligand (PD-L1 = CD274 and PD-L2), which binds to programmed cell death protein 1 (PD-1), expressed by T-cells, subsequently promoting tolerance via induction of clonal anergy and regulatory T-cells differentiation.^16^ Plasmacytoid DCs may support the expansion of myelin-specific regulatory T-cells through HLA-DR–mediated antigen presentation, as shown in MS models. CD274 on DCs may further suppress auto-reactive T-cell responses. Additionally, pDCs upregulate CCR7, supporting their migratory capacity.^17^

By targeting DCs, DMDs may provide a viable strategy for the treatment of MS, and research on the effects of DMDs on DCs is rare. The induction of tolerogenic DCs, which possess significant therapeutic potential, has been demonstrated to counter autoimmune reactions.^18^

The National MS Society has identified more than 136 studies to evaluate the different therapeutic options for MS. The United States Food and Drug Administration has approved the following drugs for RRMS: injectables (interferon and glatiramer acetate), oral therapies (fingolimod, teriflunomide, dimethyl fumarate, siponimod, cladribine), infusion therapies (natalizumab, ocrelizumab, alemtuzumab, and rituximab), and other (azathioprine, laquinimod, cyclophosphamide, daclizumab, dalfampridine, mitoxantrone, glucocorticoids, intravenous immunoglobulin, dalfampridine).^19^

Interferon beta-1A (IFN-b-1A) decreases the frequency of relapses, progression of disability, MRI lesion count, and provides cognitive advantages.^20^ Fingolimod, which modulates the S1P receptor, sequesters naïve and memory T-cells within lymph nodes, resulting in reduced lymphocyte levels in the blood and addressing all forms of MS.^21^ Rituximab, which targets CD20+ B-cells, lessens inflammation, decreases relapses, and results in fewer new brain lesions in RRMS.^22^

The aim of our study was to evaluate the effect of DMDs such as IFN-b-1A, fingolimod and B-cell (rituximab) therapy on NFL and CHI3L1 serum levels, and some biomarkers of pDCs in patients diagnosed with RRMS.

## Methods

### Ethical considerations

This study was conducted in accordance with the ethical standards of the Declaration of Helsinki as well as national legislation and institutional guidelines. Approval for the collection and analysis of samples and clinical data was obtained from the Ethical Committee Faculty of Medicine, Assiut University, Egypt (reference number: 17200750). All subjects in this study provided written informed consent to participate. All patient data were de-identified and replaced with study codes before analysis. The coding key was stored separately and accessible only to the investigators. Electronic files were kept on password-protected computers, and physical documents were stored in locked cabinets. Only anonymized, aggregated data were used in the study and in all publications to maintain full confidentiality.

### Study settings

This study was conducted at the Clinical Pathology Department and patients were recruited from the Neurology Department, at Assiut University Hospital located inside Assiut University, Second Division, Assiut city in Upper Egypt. It consists of 11 buildings, operates 24 h and is considered one of the largest university hospitals in Egypt. The total number of beds in the university hospital is nearly 3000, of which 92% are free beds and 8% are private and economical treatment beds. The outpatient clinics receive about two million patients annually from all governorates of Upper Egypt (https://healtheg.com/en/Item/2670/Assiut-University-Hospital; https://www.aun.edu.eg/hospitals/).

### Recruitment of study participants

This cross-sectional observational study was conducted between August 2023 and July 2024, on 30 healthy controls and 44 RRMS patients who were diagnosed according to McDonald criteria 2017, evaluated by full neurological examination for patients’ staging and expanded disability status scale (EDSS); all presented with brain lesions by MRI results and more than two CSF oligoclonal bands by isoelectrofocusing were not present in paired serum samples. Patients actively receiving their DMDs were divided into three groups according to DMDs type: Group 1 (*n* = 17) received IFN-b-1A, Group 2 (*n* = 20) received fingolimod, and Group 3 (*n* = 7) received B-cell (rituximab) therapy. Patients of all ages and both sexes were included. The 44 patients were selected from 220 patients; the rest were excluded for various reasons (other MS subtypes, primary progressive MS, or secondary progressive MS, recently diagnosed; not adhering to their treatment plan; refused to participate). According to G*Power 3 software,^23^ sample size calculation in cases to examine the differences according to treatment was as follows: a minimum of 21 patients with RRMS divided into three groups: Group 1 (*n* = 7) on IFN-b-1A treatment, Group 2 (*n* = 7) on fingolimod treatment, and Group 3 (*n* = 7) on B-cell therapy (rituximab) treatment to detect an effect size of 0.3 in the mean levels of serum NFL and CHI3L1, with an error probability of 0.05, and 85% power on a one-tailed test.^24^ The study was registered at ClinicalTrials.gov (National Clinical Trial number: NCT05451069).

### Sample collection and storage

Blood samples: 2 mL of blood was collected into an ethylenediaminetetraacetic acid-containing vacutainer for flow cytometric analysis, and 3 mL was collected into a gel vacutainer and centrifuged at 2000 rpm – 3000 rpm for 15 min. The serum was separated and stored at –80 °C until the NFL and CHI3L1 assays. All procedures related to sample handling and storage were carried out according to established and routinely used methods by Assiut University laboratories of the Clinical Pathology Department, following standard operating procedures.

### Data management and analysis

Data management and analyses were conducted using SPSS version 27.0 (IBM Corporation, Armonk, New York, United States). Non-parametric data were described using the median and range, and parametric quantitative data were described using the mean and standard deviation. Qualitative data were presented as numerical values and percentages. Independent categorical variables were compared using the chi-squared test. Kruskal-Wallis tests were applied for non-normally distributed continuous data whereas one-way analysis of variance was used for normally distributed data. Spearman’s correlation was used to evaluate biomarkers, and the receiver operating characteristic curve was used to assess the specificity and sensitivity of serum NFL and CHI3L biomarkers. The *p*-value was two-tailed, with a significance set at 0.05.

### Measurement of serum levels of neurofilament light chain and chitinase 3-like 1

Serum NFL was measured by enzyme-linked immunosorbent assay using the Human Neurofilament Light Chain Kit (Uman Diagnostics AB, Tvistevägen 48C, Umeå, Sweden),^25^ and the data were represented as pg/mL. Serum CHI3L1 was measured by enzyme-linked immunosorbent assay using the Human CHI3L1 Kit (Sino Gene Clon Biotech Co., Ltd, Hangzhou City, Zhejiang, China) and the data were represented as ng/mL. The plates were read at an absorbance of 450 nm using a Stat Fax 200 (Awareness Technology, Inc., Palm City, Florida, United States).

### Flow cytometric analysis of plasmacytoid dendritic cells

Blood samples collected in ethylenediaminetetraacetic acid vacutainers were stored at room temperature (20 °C – 25 °C), stained, and analysed on the same day as blood collection using a flow cytometry system (Beckman Coulter CytoFLEX; Beckman Coulter, Brea, California, United States). After titration to estimate the proper concentration to be used, 3 µL of each monoclonal antibody was pipetted: CD303-APC (Elabscience, Houston, Texas, United States), Anti-HLA-DR-APC-Alexa Fluor 750, CD274-PC7 (PD-L1) and CD197-PE (CCR7) (Beckman Coulter, Marseille, France), and 100 µL of well-mixed ethylenediaminetetraacetic acid blood samples was added to the monoclonal antibodies. Fluorescence minus one control was used to determine the positivity for each monoclonal antibody. Positive regions were set above 10^4^ on the log scale of the dot plot for CD303, and above 10^3^ for HLA-DR, CD274, and CCR7.^26^

Acquisition and analysis were conducted using the Beckman Coulter CytoFLEX, with data processed by CytExpert 2.5 software (Beckman Coulter, Inc., Brea, California, United States), ensuring that 50 000 cells were acquired. The cell count was adjusted to 3 × 10^4^ – 10 × 10^4^. Negative unstained control was initially used to establish negative and positive population locations. To establish the upper limit for the background signal and delineate positive populations, four fluorescence minus one control tubes were prepared, each excluding one fluorochrome because of the low expression of pDCs. Forward scatter (FSC-*x*-axis) versus side scatter (SSC-*y*-axis) was displayed on a dot plot. Each monoclonal antibody was represented by its percentage and mean fluorescence intensity.

Sequential gating was conducted to differentiate pDCs and their tolerogenic and migratory functions, based on light scatter and antigenic profiles. The hierarchical analysis gating sequence was made as follows: We first gated the mononuclear cells (P1) from the whole leukocytes, then gated P2 derived from P1 to define pDCs by surface marker (CD303-APC vs SSC-A). From P2, the second identification marker (HLA-DR) was plotted on FSC-A (HLA-DR-APC-Alexa Fluor 750) versus SSC-A (CD-303-APC). To assess the tolerogenic function of pDCs, antigen-based gates were displayed from P2 on FSC-A (CD274-PC7) versus SSC-A (CD303-APC). To assess the migratory function of pDCs, antigen-based gates were displayed from P2 on FSC-A (CCR7-PE) versus SSC-A (CD303-APC).

## Results

The 44 MS patients were subdivided by treatment type as follows: 17 patients on IFN-b-1A, 20 patients on fingolimod, and 7 patients on B-cell therapy (rituximab).

### Demographic characteristics of the three multiple sclerosis treatment groups

There were no significant differences in these demographic variables between the patients and controls.

As shown in Table 1, in terms of marital status(*p* = 0.145), sex (*p* = 0.159), and job (*p* = 0.078), no significant differences were found between the DMDs groups.

**TABLE 1:** Demographic characteristics of the three multiple sclerosis treatment groups, Upper Egypt, between August 2023 and July 2024.

Variable	Category	Group 1 (IFN-b-1A)				Group 2 (fingolimod)				Group 3 (B-cell therapy)				*p*-value†
		*n*	%	Range	Mean ± s.d.	*n*	%	Range	Mean ± s.d.	*n*	%	Range	Mean ± s.d.	
Marital status†		-	-	-	-	-	-	-	-	-	-	-	-	0.145
	Single	7	41.2	-	-	3	15.0	-	-	1	14.3	-	-	
	Married	10	58.8	-	-	17	85.0	-	-	6	85.7	-	-	
Sex†		-	-	-	-	-	-	-	-	-	-	-	-	0.159
	Female	16	94.1	-	-	14	70.0	-	-	6	85.7	-	-	
	Male	1	5.9	-	-	6	30.0	-	-	1	14.3	-	-	
Employment†		-	-	-	-	-	-	-	-	-	-	-	-	0.078
	Employee	16	94.1	-	-	17	85.0	-	-	4	57.1	-	-	
	Housewife	1	5.9	-	-	3	15.0	-	-	3	42.9	-	-	
Age (years)‡		-	-	15–49	33.53 ± 10.4	-	-	31–59	41.65 ± 9.29	-	-	21–59	41.71 ± 13.80	0.054
Group 1 versus Group 2		-	-	-	-	-	-	-	-	-	-	-	-	0.024
Group 1 versus Group 3		-	-	-	-	-	-	-	-	-	-	-	-	0.090
Group 3 versus Group 2		-	-	-	-	-	-	-	-	-	-	-	-	0.989

s.d., standard deviation; IFN-b-1A, interferon beta-1A.

† , Chi-square test was used to compare frequency between groups.

‡ , One-way analysis of variance was used to compare means with post-hoc test for pairwise comparisons with Tukey’s corrections.

For age, patients on IFN-b-1A ranged from 15 to 49 years (mean ± standard deviation: 33.53 ± 10.4), those on fingolimod from 31 to 59 years (mean ± standard deviation: 41.65 ± 9.29), and those on B-cell therapy from 21 to 59 years (mean: 41.71 ± 13.80). There is a significant difference between the three groups (*p* = 0.054). The post-hoc analysis revealed that Group 2 (fingolimod) showed higher age group than Group 1 of (IFN-b-1A) (*p* = 0.024).

### Magnetic resonance imaging of the three multiple sclerosis treatment groups

Patients on B-cell therapy had higher percentages of positive MRI brain atrophy (85.7%), MRI cord atrophy (85.7%), and MRI cord lesion (85.7%), with significant differences between groups (*p* < 0.001), as shown in Table 2.

**TABLE 2:** Magnetic resonance imaging of brain atrophy, cord atrophy, brain lesion, and cord lesion between the three multiple sclerosis treatment groups, Upper Egypt, between August 2023 and July 2024.

Variable	Group 1 (IFN-b-1A)				Group 2 (fingolimod)				Group 3 (B-cell therapy)				*p*-value†
	Positive		Negative		Positive		Negative		Positive		Negative		
	*n*	%	*n*	%	*n*	%	*n*	%	*n*	%	*n*	%	
MRI brain atrophy	0	0	17	100	9	45	11	55	6	85.7	1	14.3	< 0.001*
MRI cord atrophy	0	0	17	100	7	35	13	65	6	85.7	1	14.3	< 0.001*
MRI brain lesion	16	94.1	1	5.9	20	100	0	0	7	100	0	0	0.545
MRI cord lesion	3	17.6	14	82.4	7	35	13	65	6	85.7	1	14.3	0.007*

MRI, magnetic resonance imaging; IFN-b-1A, interferon beta-1A.

* , *p*-value was significant if < 0.05.

† , Chi-square test was used to compare frequency between groups.

### Measurements of serum neurofilament light chain and chitinase 3-like 1

Neurofilament light chain (pg/mL) in the control group ranged from 1.60 pg/mL to 14.00 pg/mL, with a median of 5.60 pg/mL, and from 1.60 pg/mL to 258.00 pg/mL, with a median of 12.20 pg/mL, in the case group, indicating higher levels in the case group (*p* < 0.001). Chitinase 3-like 1 (ng/mL) in the control group ranged from 3.00 ng/mL to 21.00 ng/mL, with a median of 5.50 ng/mL, and from 3.50 ng/mL to 2156.50 ng/mL, with a median of 43.25 ng/mL, in the case group, indicating a higher level in the case group (*p* < 0.001).

Among the three treatment groups, NFL (pg/mL) showed higher levels in the B-cell therapy group, with a range of 37.2 pg/mL to 258.0 pg/mL, a median of 76.80 pg/mL, compared with IFN-b-1A (*p* < 0.001) and fingolimod (*p* = 0.005). Chitinase 3-like 1 (ng/mL) showed higher levels in the B-cell therapy group, with a 61.00 ng/mL to 2156.50 ng/mL range, a median of 396.00 ng/mL, and a *p*-value of 0.001 and 0.015 compared to IFN-b-1A and fingolimod, as shown in Table 3.

**TABLE 3:** Serum neurofilament light chain and chitinase 3-like 1 biomarkers between the three multiple sclerosis treatment groups, Upper Egypt, between August 2023 and July 2024.

Variable	Group 1 (IFN-b-1A)		Group 2 (fingolimod)		Group 3 (B-cell therapy)		*p*-value
	Range	Median	Range	Median	Range	Median	
**NFL (pg/mL)** †	1.60–20.4	8.80	4.80–70.80	12.00	37.20–258.00	76.80	< 0.001*
Group 1 versus Group 2‡	-	-	-	-	-	-	0.312
Group 3 versus Group 2‡	-	-	-	-	-	-	0.005*
Group 1 versus Group 3‡	-	-	-	-	-	-	< 0.001*
**CHI3L1 (ng/mL)** †	3.50–72.00	34.00	3.50–794.00	40.75	61.00–2156.50	396.00	0.002*
Group 1 versus Group 2‡	-	-	-	-	-	-	0.876
Group 3 versus Group 2‡	-	-	-	-	-	-	0.015*
Group 1 versus Group 3‡	-	-	-	-	-	-	0.001*

NFL, neurofilament light chain biomarker; CHI3L1, chitinase 3-like 1 biomarker; IFN-b-1A, interferon beta-1A.

* , *p*-value was significant if < 0.05.

† , Kruskal Wallis test was used to compare means with ‡;

‡ , post-hoc test for pairwise comparisons with Tukey’s corrections.

**FIGURE 1:**
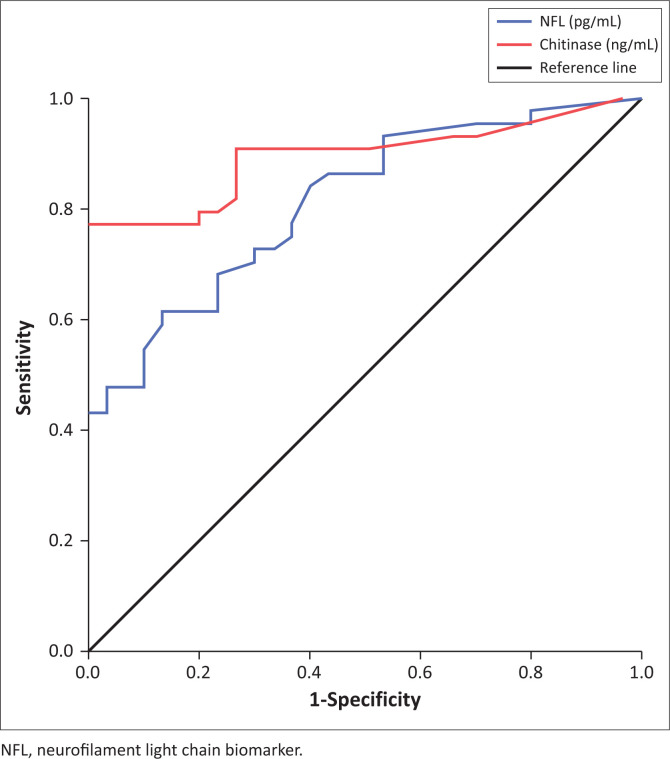
Receiver operating characteristic curve of serum neurofilament light chain and chitinase 3-like 1 biomarkers, Upper Egypt, between August 2023 and July 2024.

Neurofilament light chain levels at a cutoff point ≤ 6.600 pg/mL showed 84.1% sensitivity and 60.0% specificity for measuring the response of DMDs in MS, with an area under the curve of 0.814, 95% confidence interval (CI): 0.719 to 0.909 and a *p*-value < 0.001. Chitinase 3-like 1 levels at a cut-off point ≤ 10.25 ng/mL showed 90.9% sensitivity and 73.0% specificity for measuring the response of DMDs in MS, with an area under the curve of 0.895, 95% CI: 0.821 to 0.969 and a *p*-value < 0.001, as shown in Table 4 and Figure 1.

**TABLE 4:** Sensitivity, specificity, and cut-off value of serum levels neurofilament light chain and chitinase 3-like 1 biomarkers, Upper Egypt, between August 2023 and July 2024.

Variable	AUC	95% CI	Cut-off point	Sensitivity (%)	Specificity (%)	*p*-value†
NFL (pg/mL)	0.814	0.719–0.909	≤ 6.60	84.1	60.0	< 0.001*
CHI3L1 (ng/mL)	0.895	0.821–0.969	≤ 10.25	90.9	73.0	< 0.001*

AUC, area under curve; CI, confidence interval; ROC, receiver operating characteristic; NFL, neurofilament light chain biomarker; CHI3L1, chitinase 3-like 1 biomarker.

* , *p*-value was significant if < 0.05.

† , ROC curve analysis.

### Expanded disability status scale and flow analysis between the three multiple sclerosis treatment groups

As shown in Table 5, EDSS in patients on IFN-b-1A ranged from 1.0 to 3.0, with a median of 2.0; in those on fingolimod, it ranged from 2.0 to 6.0, with a median of 3.0, and in those on B-cell therapy, it ranged from 4.0 to 7.0, with a median of 5.0, indicating a higher EDSS in the B-cell therapy group (*p* < 0.001).

**TABLE 5:** Comparison of expanded disability status scale, blood dendritic cell antigen BDCA-2 (CD303), HLA-DR, CD274, and CCR7 between the three MS treatment groups, Upper Egypt, between August 2023 and July 2024.

Variable	Group 1 (IFN-b-1A)		Group 2 (fingolimod)		Group 3 (B-cell therapy)		*p*-value
	Range	Median	Range	Median	Range	Median	
**EDSS**	1.00–3.00	2.00	2.00–6.00	3.00	4.00–7.00	5.00	< 0.001†
Group 1 versus Group 2	-	-	-	-	-	-	0.051‡
Group 3 versus Group 2	-	-	-	-	-	-	< 0.001‡
Group 1 versus Group 3	-	-	-	-	-	-	< 0.001‡
**MNCs (%)**	12.00–81.00	40.00	10.00–85.00	35.00	14.00–49.00	31.00	0.260
**BDCA-2 (CD303) (%)**	0.00–71.00	0.00	0.00–49.00	1.00	0.00–21.00	1.00	0.590
**BDCA-2 (CD303) MFI gemoetric mean**	6351.50–15 155.60	9117.70	6545.00–29 755.40	9418.50	6584.10–21 665.20	10 630.50	0.730
**HLA-DR (%)**	33.00–100.00	84.00	40.00–100.00	88.00	0.00–89.00	72.00	0.150
**HLA-DR MFI geometric mean**	2500.00–152 653.00	60 179.50	1309.50–176 313.00	58 659.00	0.00–76 836.00	15 236.20	0.056†
**CD274 (%)**	0.00–79.10	20.00	0.00–91.00	9.00	510.00–100.00	51.00	0.330
**CD274 MFI geometric mean**	0.00–7475.20	1637.20	0.00–44 281.00	1895.40	0.00–104 441.40	3107.60	0.383
**CCR7 (%)**	0.00–92.20	32.00	0.00–99.00	30.00	0.00–73.00	43.00	0.720
**CCR7 MFI geometric mean**	0.00–4331.40	2331.50	1078.20–9978.30	2079.60	0.00–7982.00	2337.60	0.970

EDSS, expanded disability status scale; BDCA-2, blood dendritic cell antigen; MNC, mononucluear cells; MFI, mean fluorescence intensity; HLA-DR, human leukocyte antigen-DR; CD, cluster of differentiation; CCR, chemokine receptor; IFN-b-1A, interferon beta-1A.

† , Kruskal Wallis test was used to compare means with ‡;

‡ , post-hoc test for pairwise comparisons with Tukey’s corrections.

The percentages and mean fluorescence intensity of MNC, CD303, HLA-DR, CD274, and CCR7, except for the HLA-DR mean fluorescence intensity mean (*p* = 0.056), showed no significant difference between the three DMDs groups.

### Correlation of serum neurofilament light chain and chitinase 3-like 1 with other study parameters

Neurofilament light chain serum levels showed no significant correlation with CD303 (*r* = 0.045, *p* = 0.351) and CD274 (*r* = 0.189, *p* = 0.054); however, they showed a positive significant correlation with CHI3L1 serum levels (*r* = 0.592, *p* ≤ 0.001), EDSS (*r* = 0.560, *p* ≤ 0.001), and CCR7% (*r* = 0.276, *p* ≤ 0.017). CHI3L1 levels showed no significant correlation with CD303 (*r* = 0.182, *p* = 0.061) and CD274 (*r* = 0.130, *p* = 0.135), but they had a positive significant correlation with NFL levels (*r* = 0.592, *p* ≤ 0.001), EDSS (*r* = 0.422, *p* ≤ 0.004), and CCR7% (*r* = 0.248, *p* ≤ 0.033).

## Discussion

Neuronal and axonal destruction are among the pathological characteristics of MS.^27^ Research indicates that high-efficacy DMDs can be introduced to reduce disease activity, prevent disability, and help regulate disease progression.^28,29,30^

The pathophysiology of RRMS changes over time, and as more treatment choices become available, there is a need for more methods to measure disease activity and response to therapy, such as biomarkers in body fluids.^31,32^ Neurofilament light chain, an axonal protein, and CHI3L1, a glial glycoprotein in the CNS, have been shown to serve as biomarkers for inflammation and axonal injury in MS.^33^ Enhanced comprehension of their reaction to DMDs may facilitate their incorporation into clinical uses and treatment monitoring.^34^

To our view, investigations of the combined serum levels of NFL and CHI3L1 and the role of tolerogenic pDCs in response to DMDs for MS treatment are rare.

The age and gender profile of the patients in this study are consistent with previous reports in the literature, for example Zakaria et al., who reported in 2016 that the mean age of their patients was 31.87 ± 8.7, and 72% were female.^35^ We reported that the fingolimod group had higher mean ages than the IFN-b-1A group. In contrast to fingolimod, which is reserved for more advanced instances that typically worsen as patients age, the majority of female RRMS patients in our study prefer to use IFN-b-1A more frequently at lower ages during the childbearing period. Macaron et al. in 2023 reported that as patients age, they may be exposed to more DMDs with varying mechanisms of action.^36^

We found that the employment rates in the B-cell therapy group were 57.1%, 85% for fingolimod, and 94.1% for IFN-b-1A. The lower employment rate observed in the B-cell therapy group may be attributable to their significantly higher EDSS score, which ranged from 4 to 7, with a median of 5. The association between unemployment and physical disability in MS has been widely reported,^37^ with 82% of individuals employed at an EDSS of 0, but only 25% employed at an EDSS of 6.5.^38^

All patients in our study underwent MRI before treatment initiation. The B-cell therapy group demonstrated a higher percentage of brain atrophy, cord atrophy, and cord lesions compared to the other treatment groups. IFN-b-1A was linked to the lowest rates of MRI lesions and atrophy, which could be attributed to its classification as a first-line treatment for the early stages of RRMS. In contrast, the increased percentage of brain atrophy and spinal cord lesions associated with B-cell therapy may result from its application in more advanced disease stages. Multiple sclerosis patients on IFN-b-1A for a year, who exhibit at least two of three variables (relapses, increased disability, or MRI activity), are candidates for treatment modification because of disease progression risk.^39^

Our study showed that serum NFL levels at a cut-off ≤ 6.600 pg/mL and CHI3L1 levels ≤ 10.25 ng/mL were elevated in MS patients compared to non-MS controls, which was consistent with previous findings.^40,41,42^ As axonal injury increases, the progression of disability becomes more severe. Serum NFL was significantly correlated with EDSS (*r* = 0.560, *p* ≤ 0.001), aligning with larger studies involving 814 and 607 patients, which found a weak yet significant association between EDSS and serum NFL levels, showing a 12% and 8% NFL increase per EDSS step.^43,44^ Furthermore, serum NFL is correlated with CHI3L1, suggesting that glial activation is associated with axonal injury and the progression of disability in MS.^45^

Comparing the serum NFL levels across the three treatment groups, we observed the highest levels in the B-cell therapy group compared with fingolimod (*p* = 0.005) and IFN-b-1A (*p* < 0.001). This could be attributed to the association of NFL levels with MRI findings and higher EDSS in this group, as short-term prognostic studies^42,44,45,46^ have demonstrated that NFL levels correlate with EDSS deterioration, relapse frequency, and brain atrophy measured by MRI.

Chitinase 3-like 1 is a potential therapeutic target for the treatment of MS. Strategies to suppress CHI3L1 may reduce neuroinflammation, aid remyelination, and protect axons.^47^ Our results showed that serum CHI3L1 levels were higher in the B-cell therapy group compared with IFN-b-1A and fingolimod groups, with a significant positive correlation with EDSS (*r* = 0.422, *p* ≤ 0.004), contrasting with the findings of a study that reported that CHI3L1 levels were not associated with DMD type but rather with long-term DMD use.^47^ Being a proinflammatory marker secreted by various immune cells such as macrophages, synoviocytes, neutrophils, and endothelial cells, it is anticipated that it plays a role in the pathogenesis of MS. However, we cannot conclude that IFN-b-1A and fingolimod directly inhibit its levels in comparison to B-cell therapies; their effects may be more associated with the stages of disability and inflammation in the B-cell therapy group. Larger studies on CHI3L1 serum levels are needed for it to be evaluated as a biomarker for DMDs response.

Dendritic cells are essential for eliciting either an inflammatory or tolerogenic response. Disease-modifying drugs can significantly affect DC function in MS. However, unlike T- and B-cells, the effects of DMDs on DCs are less well studied, although tolerogenic DC induction is recognised for its therapeutic potential in preventing autoimmune reactions.^18^

In this study, we investigated the role of pDCs in MS by examining the percentage of pDCs and their tolerogenic and migratory functions in response to three DMD groups. We found that, although there was an increase in the percentage of pDCs, which ranged from 0.0 to 71.0 in patients with MS compared to the healthy group (0.0–4.0), it was not statistically significant between the two groups or across the treatment groups. This may be due to the absence of CSF samples in our study and the fact that our patients were in the remitting stage. Previous reports have shown that pDC levels increase in the CSF and demyelinating lesions during disease exacerbations.^48^ Since pDC function differs between tissues, their blood levels may not accurately reflect their activity in lymph nodes or the CNS. As a result, sampling from peripheral blood might overlook important migratory or immunoregulatory functions of pDCs that are only observable in their native locations which would be more informative by the CSF samples. In remission and under effect of DMDs, inflammation tends to be lower, which could lead to less activation or migration of pDCs compared to periods of active disease. This inactive immune environment might obscure changes in pDC function that occur during more active disease phases.

We assessed pDC tolerogenic behaviour by assessing PD-L1 expression on pDCs and their migration by identifying CCR7. No significant difference was observed between patients with MS and controls or across treatment groups, but we found a significant positive correlation between NFL, CHI3L1, and pDCs’ migratory function. Plasmacytoid DCs, constituting only 0.3% – 0.5% of peripheral blood, are challenging to study, and their role in MS is still unclear.^49^

The limitation of this study was obtaining CSF samples which could reveal localised immune signatures not captured peripherally and provide more sensitive CNS data alongside peripheral samples to better analyse pDC, NFL and CHI3L1 correlation, but our patients declined this procedure. Further studies are necessary to examine the long-term clinical outcomes of various DMDs and to establish NFL and CHI3L1 levels as potential drug response biomarkers. The small B-cell therapy cohort likely reflects its use mainly in highly active RRMS, initial use in primary progressive MS, and limited use in stable RRMS, according to the protocol of the Committee of National Egyptian guidelines, Ministry of Health and Neurology Scientific Committee. Few studies have explored the role of pDCs in MS or the effects of DMDs on these cells. Modifying pDCs could emerge as a strategy to enhance immune control in MS, although their specific role in MS requires further clarification, particularly regarding the effects of DMDs.

### Conclusion

In conclusion, DMDs type (IFN-b-1A, fingolimod, and B-cell therapy) impacts NFL and CHI3L1 serum levels of RRMS patients of Upper Egypt and relates to their clinical data, but has no diverse impact on the migratory and tolerogenic function of pDCs.
